# A Fast and Cost-Effective Genotyping Method for CRISPR-Cas9-Generated Mutant Rice Lines

**DOI:** 10.3390/plants12112189

**Published:** 2023-05-31

**Authors:** Abdugaffor Ablazov, Abrar Felemban, Justine Braguy, Hendrik N. J. Kuijer, Salim Al-Babili

**Affiliations:** 1 Center for Desert Agriculture (CDA), The BioActives Laboratory, Biological and Environmental Sciences and Engineering Division, King Abdullah University of Science and Technology (KAUST), Thuwal 23955-6900, Saudi Arabia; 2 The Plant Science Program, Biological and Environmental Science and Engineering Division, King Abdullah University of Science and Technology (KAUST), Thuwal 23955-6900, Saudi Arabia

**Keywords:** CRISPR-Cas9, genotyping, PCR-direct, transgenicity, sanger sequencing, ZAXINONE SYNTHASE

## Abstract

With increasing throughput in both the generation and phenotyping of mutant lines in plants, it is important to have an efficient and reliable genotyping method. Traditional workflows, still commonly used in many labs, have time-consuming and expensive steps, such as DNA purification, cloning and growing *E. coli* cultures. We propose an alternative workflow where these steps are bypassed, using Phire polymerase on fresh plant tissue, and ExoProStar treatment as preparation for sequencing. We generated CRISPR-Cas9 mutants for ZAS (ZAXINONE SYNTHASE) in rice with two guide RNAs. Using both a traditional workflow and our proposed workflow, we genotyped nine T1 plants. To interpret the sequencing output, which is often complex in CRISPR-generated mutants, we used free online automatic analysis systems and compared the results. Our proposed workflow produces results of the same quality as the old workflow, but in 1 day instead of 3 days and about 35 times cheaper. This workflow also consists of fewer steps and reduces the risk of cross contamination and mistakes. Furthermore, the automated sequence analysis packages are mostly accurate and could easily be used for bulk analysis. Based on these advantages, we encourage academic and commercial labs conducting genotyping to consider switching over to our proposed workflow.

## 1. Introduction

Rice (*Oryza sativa*) is amongst the three most important crops worldwide [1]. Moreover, given its role as a model plant for other cereals [2], deciphering rice molecular mechanisms is crucial for ensuring food security. To uncover gene functions, scientists rely on generating loss-of-function mutations as a common approach [3]. The discovery of the clustered regularly interspaced short palindromic repeats (CRISPR) together with CRISPR-associated protein 9 (Cas9) and their implementation as a genome editing tool revolutionized biology. Briefly, the CRISPR-Cas9 molecular mechanism makes use of the Cas9 ability to generate DNA double-strand breaks (DSB), when paired/hybridized with RNA. This RNA–DNA-directed cleavage is targeted to a specific DNA site by a short (usually 20-nucleotide long) complementary RNA sequence named single guide RNA (gRNA or sgRNA), enabling the non-precise, non-homologous end-joining repair mechanism of the host cell to generate mutated versions [4].

The CRISPR-Cas9 technology, being optimized for usage in plant systems, was first applied to edit the rice genome in 2013 [5,6]. Combined with well-detailed protocols for rice transformation, such as the one of [7], CRISPR-Cas9-mediated rice engineering rapidly became the technology of choice for precise genome editing and functional studies in laboratories all over the world [8,9]. The process from mutant generation to the precise identification of the mutation is lengthy, and the CRISPR-Cas9-mediated mutagenesis efficiency can vary according to the DNA target site [10,11]. Therefore, for a single line, it is common to genotype a minimum of 10–20 rice transformants, in search of homozygous individuals, following this three-step pipeline: (i) DNA extraction, (ii) DNA amplification and (iii) DNA preparation for sequencing. Briefly, one first extracts the rice genomic DNA before amplifying the genomic region encompassing the DNA target via polymerase chain reaction (PCR). The resulting amplicon is then purified and optionally cloned into a vector, before being sent for sequencing. The impressive number of putative transformants resulting from the genome editing required the development of inexpensive and rapid high-throughput genotyping workflows from the scientific community [9,12,13,14,15,16]. The identification of a mutation event in the transformed plants can be determined at different developmental stages: seed, calli (if induced during the transformation process) and seedling/plant.

For the development of an efficient and cost-effective workflow, we decided to target the rice *ZAXINONE SYNTHASE* (*ZAS*) gene for its biological relevance [17]. ZAS is the representative of a new clade of the plant Carotenoid Cleavage Dioxygenase family (CCDs), which produces the growth regulator zaxinone through the cleavage of apo-10′-Zeaxanthinal. The involvement of zaxinone in plant growth and development is just beginning to be understood [17,18,19], but its role in improving the growth and changing the metabolome and transcriptome of rice roots has already been demonstrated [17,19]. Moreover, recent studies have shown that ZAS and it is homologue ZAS2 regulate arbuscular mycorrhiza (AM) fungi symbiosis in rice [20,21].

Here, we propose a new workflow combining two kits, the Phire Plant Direct PCR kit (Thermo Scientific, Vilnius, Lithuania) and the illustra ExoProStar kit (Cytiva US78210), for faster, cheaper and easier-to-perform rice genotyping. In addition, we tested various freely available online software suites used for deconvoluting the sequencing chromatograms from the direct sequencing of PCR products. The use of this new work flow allows plant researchers to process one plant sample in 2 h before sending it for sequencing instead of three days, while also reducing the material costs by 97%.

## 2. Results and Discussion

### 2.1. Direct PCR Amplification from Plant Tissues Is as Efficient as from Extracted DNA

With increasing throughput in both the generation and phenotyping of mutant lines in plants, it is important to have an efficient and reliable genotyping method. PCR-based target DNA detection is a common method in plant research to validate the transgenicity and screen the mutation types, particularly CRISPR-Cas9-induced mutation. Target DNA amplification from plant tissues traditionally requires a DNA extraction step before PCR. This extraction may require a high-priced kit or the usage of harmful compounds, such as chloroform, and is time consuming [22,23]. However, the DNA target can be amplified directly from the plant tissue without DNA extraction. For example, Bellstedt reported a method where the target DNA can be directly amplified from both nuclear and plastid DNAs in a wide variety of vascular plants [24]. Similarly, Yang developed a lysis buffer that can be used to digest the plant leaves, which allows the production of a suitable template for direct PCR amplification [25]. However, many of these methods still require laborious and time-consuming pretreatments, which entail the mechanical disruption of the plant tissue and buffer preparation [26]. Direct PCR amplification kits are now commonly used for model plant species such as *Arabidopsis Thaliana*. We compared three of the most widely used kits (Phire Plant Direct PCR Kit, KAPA3G, Foregene) in terms of the price, DNA target amplification size and inclusive extra components (Supplemental Table S1). Among them, we choose the Phire Plant Direct PCR Kit (Thermo Scientific) due to the low cost; it comes with extra components including a digestion buffer, DNA ladder, nuclease-free water and control primers (Supplemental Table S1). Next, we asked the question of whether direct PCR amplification using the Phire Plant Direct PCR Kit from tissues is efficient and good enough for detecting CRISPR/Cas9-generated mutations in rice.

**Figure 1:**
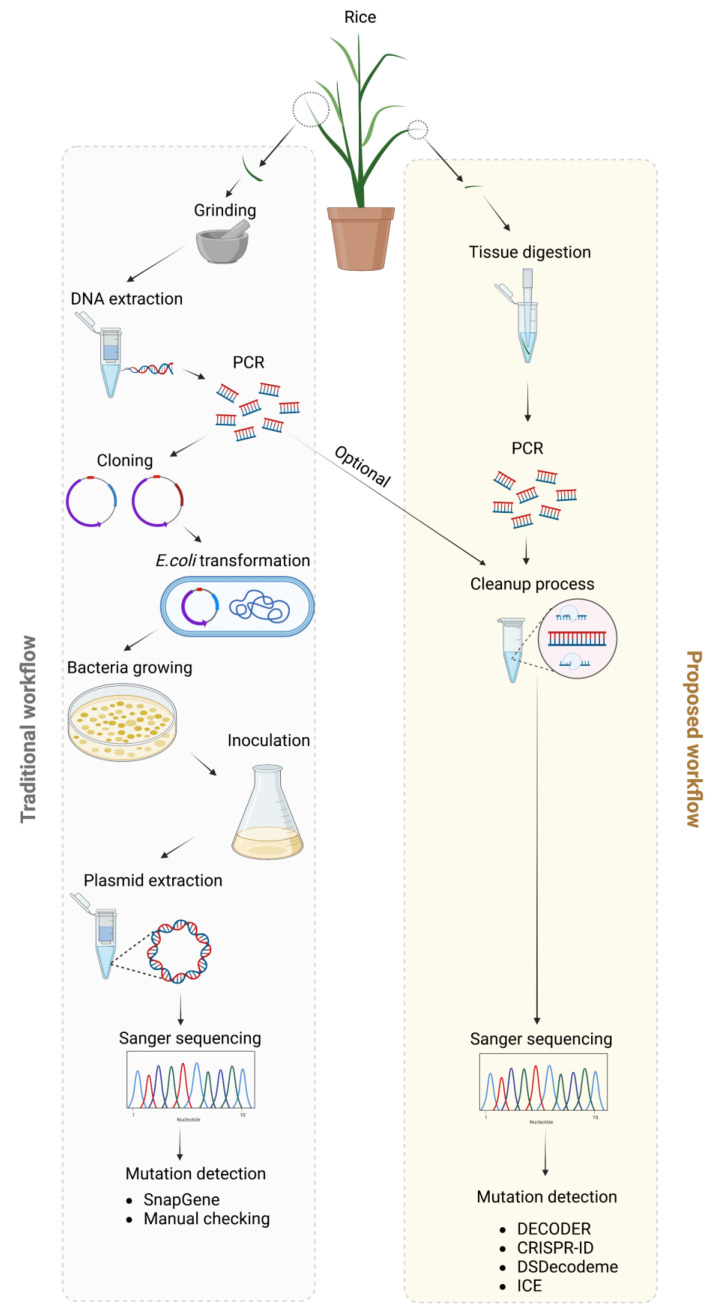
Old genotyping workflow versus proposed workflow. The old workflow consists of DNA extraction, PCR, cloning into pJet, *E. coli* transformation and growth, miniprep and sequencing. The proposed workflow shortens that to expedited DNA extraction, PCR, cleanup and sequencing. The Sanger sequencing data can be analyzed using various deconvoluting tools. Figure created using Biorender (https://biorender.com/).

**Figure 2:**
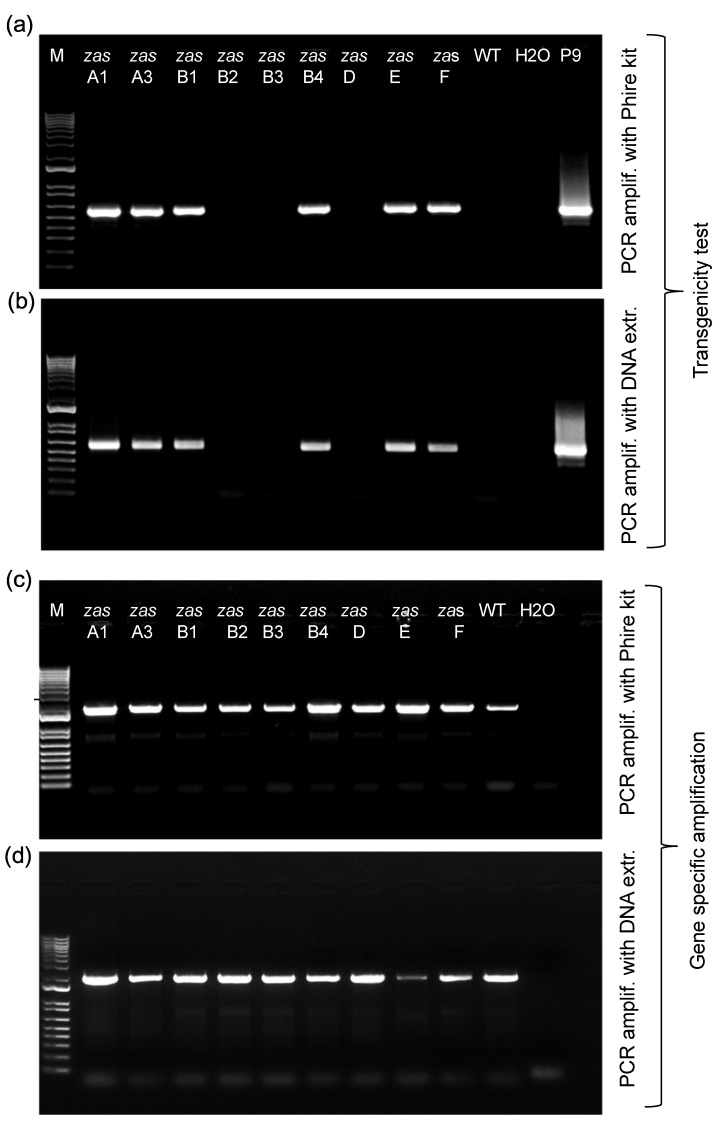
Results of PCR using the old and the proposed workflow. (**a**) Transgenicity test using the old workflow. (**b**) Transgenicity test using the proposed workflow. (**c**) Amplification before the mutagenicity test using the old workflow. (**d**) Amplification before the mutagenicity test using the proposed workflow.

Rice mutants were generated using CRISPR-Cas9 and two guide RNAs targeting exon 4 and 6 of *OsZAS*. Nine T1 mutant lines, along with wild-type plants included as a control, were selected to analyze their transgenicity and potential mutations in the *ZAS* gene. For genotyping, we used the traditional workflow, which includes DNA extraction, PCR, cloning into pJet, *E. coli* transformation and growth, miniprep and sequencing (Figure 1). In parallel, we tested our proposed workflow, which shortens the process to expedited DNA extraction, PCR, cleanup and sequencing (Figure 1). Initially, we determined the transgenicity, using primers flanking 650 bp of the gRNA encoding section of the CRISPR cassette. Six out of nine lines showed an amplicon using both methods (Figure 2a,b). This indicates that the insertion of the CRISPR cassette was successful in the parent plants of these six lines, and that the CRISPR-Cas9 cassette was not segregated out yet in these six plants. For the other three lines, it can be assumed that the CRISPR cassette was lost between T0 and T1. The quality of the results of both workflows is identical, showing that determining transgenicity directly from tissues is as efficient as from isolated DNA samples. Next, the target sites of the *OsZAS* gene, a 2000 bp section, was amplified and this was successful for all lines in both workflows (Figure 2c,d). This result suggests again that direct PCR amplification from tissues is reliable, despite skipping the DNA isolation step and the relatively large amplicon. The PCR product was then cleaned up using the ExoProStar kit and sent for Sanger sequencing. Previously, Bell demonstrated that ExoSAP-IT (or ExoProStar kit) treatment requires only one pipetting step, making it simpler to clean up multiple samples simultaneously [27]. In contrast, other methods (ethanol precipitation, column chromatography and gel purification) entail several steps and are impractical when dealing with a large number of samples. Additionally, these methods are often associated with sample loss and the low recovery of the PCR product, which can result in a base miscall in the sequencing data.

Although we only demonstrate the usage of this workflow in rice, it should be applicable to most plant species without extensive modifications. For those species that contain high levels of secondary metabolites that may impede polymerase activity, it may be necessary to include additional purification steps before the initial PCR. Besides this, we collected the leaf samples from four-week-old rice plants to ensure ample tissue for both the traditional and proposed workflows. At this stage, the plants are typically robust and can withstand minor damage caused by leaf cutting without affecting their growth. However, if samples need to be collected from young or unhealthy plants resulting from specific gene knockouts, we recommend using the line or cut tip for sample collection as described in [28] to minimize the impact on plant growth and development.

### 2.2. Free Online, Automatic Analysis of CRISPR-Induced Mutations Is Mostly Accurate

**Figure 3:**
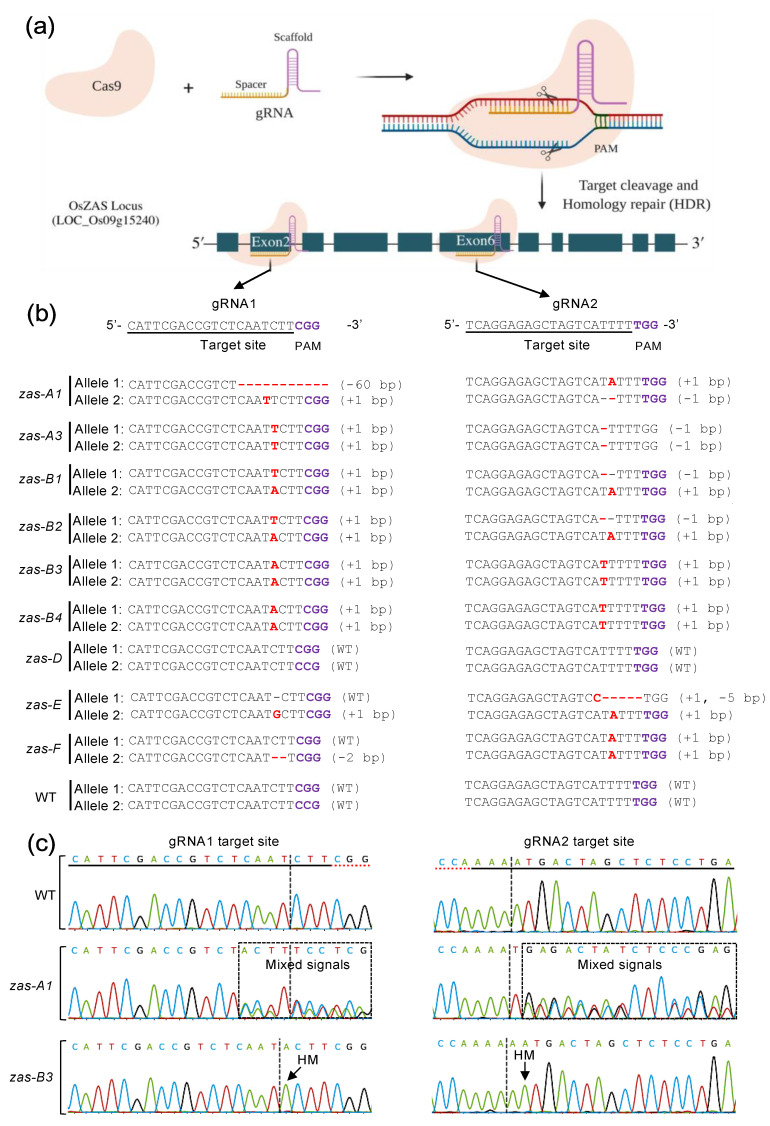
CRISPR-Cas9 targeting *OsZAS* and leading to mutations. (**a**) The Cas9 enzyme combined with a gRNA cleaves specific DNA target sites in the *ZAS* gene specified by the spacer part of the gRNAs, leading to targeted mutations. (**b**) Sequencing results of all lines for target 1 and target 2. The mutation events were identified with pJet cloning. (**c**) Chromatograms of sequenced direct PCR products of selected lines. For chromatograms of the remaining lines, see Figure S1. Dashed rectangles represent mixed signals; HM, homozygote.

CRISPR/Cas9-mediated gene editing usually generates an insertion, substitution or deletion of nucleotides within the targeted site. Moreover, these mutations can be present and identical in both (homozygous) or occur in one (heterozygous) of the two homologous chromosomes (homozygous) (Figure 3a,b). A further possibility is the introduction of different mutations on each of the homologous chromosomes (biallelic). The direct sequencing of PCR products containing such heterozygous and biallelic mutations results in superimposed sequencing chromatograms (Supplementary Figure S1; Figure 3c). For example, the direct PCR product of *zas-A1*, *B1*, *B2* and *E* in both gRNA1 and gRNA2 sites showed overlapped and mixed signals in the mutation sites (Supplementary Figure S1; Figure 3c), indicating that these lines are heterozygous. On the other hand, *zas-A3*, *B3* and *B4* showed a normal signal in both target sites, which suggests that these lines have identical mutations in both alleles. Recently, various tools have been developed to identify the CRISPR-targeted gene editing site by analyzing the chromatogram trace files of Sanger sequenced, direct PCR products [29,30,31,32]. These web-based software tools are freely accessible and allow the simultaneous analysis of multiple chromatogram trace files in a short time (2–8 min), depending on the tool’s capacity and sample size. In this study, we assessed the most widely used web-based tools, such as DSdecode (Degenerate Sequence Decode), CRISPR-ID (detecting CRISPR mediated indels by Sanger sequencing), ICE (Inference of CRISPR Edits) and DECODR (Deconvolution of Complex DNA Repair). In total, we submitted the chromatogram trace files (abi file format) of 20 samples online, following the instructions of each software suite. We recorded the results from all tools (Table 1).

In parallel, and according to the traditional genotyping method, we identified the mutations of transgenic lines from the single allele clones. For this purpose, we used around 10 clones for each gRNA target site of *OsZAS* to detect the homozygous, heterozygous and biallelic mutations (Figure 3b). Next, we compared these results with the data obtained from the abovementioned tools. The sequencing results were mostly identical for both the direct PCR product and single allele cloning approaches, with the introduced mutations being mostly a 1 bp insertion/deletion and rarely large deletions (Figure 3b). According to single cloning, several mutant lines were homozygous, such as *zas-A3*, *B3* and *B4* at both targets and *F* at target 2, while *A1*, *B1*, *B2*, *E* and *F* were heterozygous or biallelic at both target 1 and 2, except *F*, which was only heterozygous at target 1; *D* remained WT (Figure 3b).

**Figure 4:**
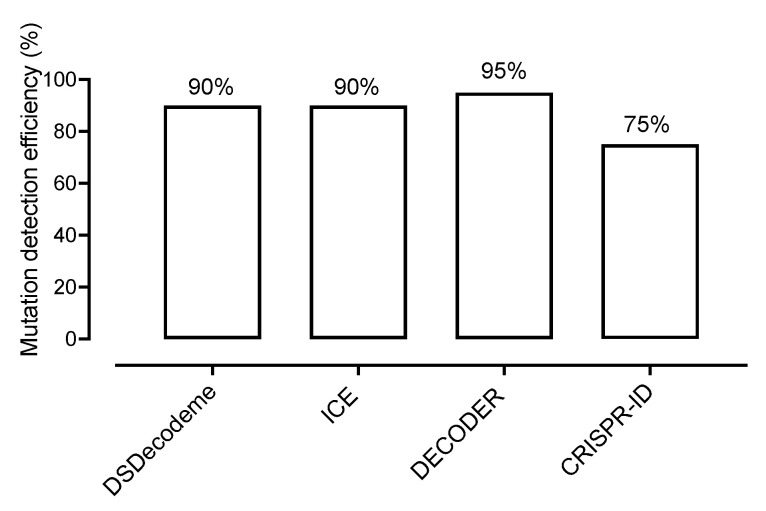
Mutation detection efficiency of the different software suites tested.

Next, we assessed the performance of each automatic decoding software program. Among all four web-based tools, DECODR was able to identify all types of mutations within all of the gRNA target sites, except one target site (gRNA1) of *zas-F* where only one allele was correctly detected as compared to pJet cloning (Table 1). The second most effective software suites were DSdecodeM and ICE, which successfully deciphered 18 mutations out of 20 (Table 1). Interestingly, both software programs failed, like DECODR, to detect the second allele mutation in the *zas1-F* line (Table 1). This suggests that the PCR product might not be well sequenced or that the algorithms of these deconvoluting suites still need to be improved to separate the mixed signals of complicated sequences. DSdecode failed to detect the gRNA2 site of *zas-E,* while ICE could not resolve one of the alleles of the gRNA1 site in *zas-A,* which showed a large deletion (Table 1). The ICE software resulted in 12 different indel types, including Wt alleles in *zas-A* lines, suggesting that this software is ineffective to detect the large deletions. DSdecode failed to detect the mutation in *zas-E*, although this program successfully deciphered the large deletion in *zas-A1.* Nevertheless, the ICE software successfully identified the indel in *zas-E.* This shows that both software suites might complement each other. CRISPR-ID was the less effective software in terms of mutation detection, as it failed to detect 5 out of 20 targets. In summary, the most accurate software was DECODR, followed by DSdecode and ICE, and least accurate was CRISPR-ID as compared to the sequencing of a single clone (Figure 4). The above result shows that decoding software is mostly efficient and accurate; however, some are currently more accurate than others. Therefore, we recommend to use at least two different software suites for deconvolution to obtain the most accurate results.

### 2.3. Proposed Workflow Much Faster and Cheaper

**Figure 5:**
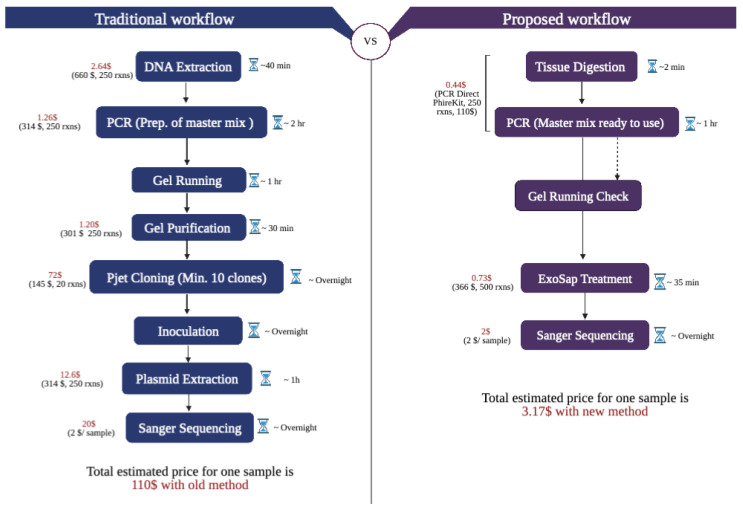
Detailed time and cost analysis of the old workflow vs. the proposed workflow. The old workflow will take 3 days and requires USD 110 per plant. The proposed workflow takes only 1 day and costs USD 3.27 per plant. The cost of the materials and reagents are presented based on purchase value in our lab at KAUST. Figure created using Biorender (https://biorender.com/).

We compared the traditional and our proposed workflow in terms of time and cost. The total time required from taking plant samples to sequencing the results for the old workflow is 3 days, because it involves *E. coli* transformation and inoculation (Figure 5), while our proposed workflow only takes 1 day (Figure 5). In addition, the amount of work to be conducted on day 1 is also reduced from over 4 h to just over 2 h. Next, we calculated and compared the cost of genotyping per plant between the regular and proposed workflow, based on the available kits and consumables in our lab. The total cost of genotyping per plant is reduced by 97% using the proposed workflow (Figure 5). The majority of the costs of the old workflow is in the pJet cloning, which is responsible for 65% of the costs per plant, and the need to send multiple samples per plant for sequencing, which is 18% of the costs per plant genotyped.

## 3. Material and Methods

### 3.1. CRISPR Targets and Vector Construction

The CRISPR-Cas9 genome-editing technique was used in the cultivar *Oryza sativa* L. ssp. *Japonica* cv. Nipponbare for targeting the *LOC_Os09g15240* gene, which encodes the enzyme ZAXINONE SYNTHASE (ZAS) [17]. Two different gRNAs were designed to target exon 2 (5′-CATTCGACCGTCTCAATCTTCGG-3′) and exon 6 (5′-TCAGGAGAGCTAGTCATTTTTGG-3′). The gRNA sequences were designed using the CRISPR-PLANT database (www.genome.arizona.edu/crispr/ (accessed on 5 October 2018). The gRNA spacers were fused to a tRNA sequence for the synthesis of polycistronic tRNA-gRNA (PTG), which was amplified from a pGTR plasmid. The plasmid was built using *FokI* and Golden Gate assembly, before being integrated into the *BsaI* pre-digested, backbone pRGEB32 plasmid [33]. The pRGEB32 binary vector was used for the stable expression of gRNA along with *Cas9* in the plant. The gRNA cassette was under the control of the rice U3snoRNA promoter (*OsU3p*), while the expression of the *Cas9* gene was driven by the rice ubiquitin promoter (*OsUBIp).* The pRGEB32 construct was then introduced into the *Agrobacterium tumefaciens* strain *EHA105* competent cells via electroporation.

### 3.2. Rice Transformation

Rice transformation was performed as previously described [34]. Briefly, rice calli, induced from Nipponbare mature seeds, were transformed with plasmid-containing *Agrobacterium* and kept in a co-cultivation medium at 25 °C in darkness for 3 days. The calli were transferred to two selection media containing hygromycin for selection and timentin as an antibacterial agent. The plates were kept for 14 days at 32 °C in the light. In order to induce shoot growth, the calli were transferred to regeneration media and kept for 14 days at 32 °C under light conditions. Once the shoots emerged, root development was promoted by transferring the shoots onto rooting media and keeping them for 14 days at 32 °C. The regenerated plants were transferred to soil prepared with half-strength modified Hoagland nutrient solution (0.18 Mm FeSO_4_.7H_2_O, 0.8 Mm MgSO_4_ × 7H_2_O, 5.6 Mm NH_4_NO_3_, 0.8 Mm K_2_SO_4_, 0.0045 Mm MnCl_2_ × 4H_2_O, 0.18 Mm Na_2_EDTA × 2H_2_O, 1.6 Mm CaCl_2_ × 2H_2_O, 0.8 Mm KNO_3_, 0.023 Mm H_3_BO_3_, 0.0003 Mm CuSO_4_ × 5H_2_O, 0.0015 Mm ZnCl_2_, 0.0001 Mm Na_2_MoO_4_ × 2H_2_O, and 0.4 Mm K_2_HPO_4_ × 2H_2_O) with an adjusted Ph of 5.8 for 1 month, after which tap water was used for watering. The plants were grown under controlled conditions in the greenhouse at 28 °C day/23 °C night.

### 3.3. Transgenicity and Mutagenicity Analysis

The transgenicity, by which we mean the insertion of the CRISPR/Cas9 cassette into the rice genome, was verified by PCR amplification, using the pRGEB32-specific primers pRGEB32-F (5′-CCACGTGATGTGAAGAAGTAAGATAAACTG-3′), and pRGEB32-R (5′-GATAGGTTTAAGGGTGATCCAAATTGAGAC-3′) targeting the gRNA encoding region on the insert. An amplicon with 650 bp length indicated the transgenicity of the analyzed line. The mutagenicity, by which we mean the introduction of mutations at the selected target sites in *OsZAS*, was tested by first amplifying the targeted area of 2000 bp by PCR using gene-specific primers, followed by Sanger sequencing. Transgenicity and mutagenicity analyses were performed using two separate workflows—the old workflow and our proposed new workflow—which are both detailed below.

### 3.4. Traditional Workflow

DNA was extracted using the Dneasy Plant Mini Kit, following the manufacturer’s instructions (Cat No. 69106, Qiagen). A small piece of rice leaf was placed in a 2 mL tube with 3 mm metallic beads and ground in liquid nitrogen using a Retsch Tissue Lyser at 28 Hz for 1 min. 400 µL of AP1 buffer (preheated for 10 min at 65 °C) and 4 µL of RNAase (100 mg/mL) was added to the ground samples and mixed by vortex, and then incubated at 65 °C for 10 min. Then, 130 µL of AP2 buffer was then added to the sample, followed by several tube inversions and incubation on ice for 5 min. The lysate was then transferred into the QIAshredder Mini spin column provided by the kit and spun down for 2 min at 14,000 rpm. The flow-through was transferred into a new tube and 1.5 volume of AP3/E buffer was added to the samples and mixed. Next, 650 µL of the mixture was transferred into the Dneasy Mini spin column and spun down for 1 min at 14,000 rpm. For the washing step, 500 µL of AW buffer was used, and the column was centrifuged for 1 min (this step was repeated one more time). The column was then transferred to a 1.5 mL microcentrifuge tube and eluted with 20 µL of elution buffer. Finally, the DNA quality and quantity was checked using a NanoDrop 2000.

For the transgenicity test, 0.1 µL of *Taq* DNA Polymerase (NEB), 10 µL of 10× PCR buffer, 0.5 µL of dNTP, 1.5 µL of MgCl_2_ and 1.5 µL (10 mM) of each of the forward (pRGEB32-F: 5′-CCACGTGATGTGAAGAAGTAAGATAAACTG-3′) and reverse primers (pRGEB32-R: 5′-GATAGGTTTAAGGGTGATCCAAATTGAGAC-3′) were added per PCR tube (0.2 mL; Neptune). Then, 1 ul of genomic DNA template and 17.5 µL of nuclease-free water were added to each tube for a final reaction volume of 25 µL. The PCR program was set as follows: initial denaturation (94 °C for 3 m), 20 cycles of denaturation (98 °C for 45 s), annealing (57 °C for 30 s) and extension (72 °C for 90 s) and a final extension at 72 °C for 10 min. The PCR product ran on 1% agarose gel.

For gene-specific amplification, 0.5 µL of Phusion DNA Polymerase (NEB), 10 µL of buffer, 1 µL of dNTPs and 2.5 µL (10 mM) of each of the forward (OsZAS-gR1-F: 5′-ACAAAATAAATGGTTTTACAATTCTGC-3′) and reverse primers (OsZAS-gR2-R: 5′-TGAGGTCAACTTTATGTTTTAGTTGAGT-3′) were added per PCR tube (0.2 mL; Neptune, Rocklin, California, United States). Finally, 2 µL of genomic DNA template and 31.5 µL of nuclease-free water were added to each tube for a final reaction volume of 50 µL. PCR was run using the C1000 Touch PCR thermal cycler (Bio-Rad) with the following conditions: initial denaturation (98 °C for 30 s), 35 cycles of denaturation (98 °C for 10 s), annealing (57 °C for 30 s) and extension (72 °C for 1 m) and a final extension at 72 °C for 10 min.

The PCR products were run on 1% agarose gel and target-band purified using a Gel and PCR Clean-Up kit (REF 740609.250, Macheney-Nagel, Dueren, Germany). Then, the purified fragments were cloned into the pJET1.2 vector, following the manufacturer’s instructions (CloneJET PCR Cloning Kit, K1232, Thermo Scientific), and transformed into TOP10 *E. coli*-competent cells using heat-shock treatment. Ten colonies grown overnight were selected and further inoculated in 5 mL LB (Luria Bertani medium) overnight, followed by DNA plasmid extraction using a QIAprep Spin Miniprep Kit (Cat. No. 27106X4, QIAGEN). To determine the putative mutation(s) at the target site, the samples were submitted for Sanger sequencing (KAUST Core Lab. Thuwal, Saudi Arabia). The chromatogram files generated by the Sanger sequencing were analyzed with SnapGene software Version 5.2.1.

### 3.5. Proposed Workflow

The DNA extraction and amplification were performed using the Phire Plant Direct kit (Thermo Fisher, Cat No. F160L). A 5 mm disc or small cut of a young leaf was placed in a 0.2 mL PCR tube and crushed with a 200 µL yellow tip in 50 µL of dilution buffer until the mixture turned greenish. Next, the samples were centrifuged for 5 s to pellet the leaf debris, and 1 µL of the supernatant was used as a template for PCR amplification. For both transgenicity and mutagenicity analyses, the PCR master mix was composed as follows: 1 µL of the supernatant as a template, 0.5 μL of each of the forward and reverse primers (the same primers as described in the old workflow), 8 µL of nuclease-free water, and 10 µL of Phire Plant Direct PCR Master Mix, which includes Phire Hot Start II DNA Polymerase, dNTPs and MgCl_2_. The annealing temperature (T_m_) of the primer pairs for PCR conditions was calculated using a Tm calculator (Thermo Scientific Web Tools, https://www.thermofisher.com/ (accessed on 10 September 2020). PCR was run using the abovementioned instrument with the following parameters: initial denaturation (98 °C for 5 m), 35 cycles of denaturation (98 °C for 6 s), annealing (30 s at 67 °C for plasmid primers and 30 s at 62 for gene specific primers) and extension (72 °C for 30 s) and a final extension at 72 °C for 1 min. After PCR amplification, 5 µL of the PCR product was analyzed on a 1% agarose gel to confirm the success of the amplification.

Then, 5 µL of the PCR product with 2 µL of the illustra ExoProStar kit (Cytiva US78210) was incubated at 37 °C for 15 min for digestion and then at 80 °C for 15 min to inactivate the ExoSAP enzyme. Then, a primer for sequencing was added and the sample was sent for Sanger sequencing (KAUST Core Lab.). The chromatogram files generated by the Sanger sequencing were analyzed using DSDecodeM (Degenerate Sequence Decode) [29], ICE (Inference of CRISPR Edits) [30], DECODR v3.0 (Deconvolution of Complex DNA Repair) [31] and CRISPR-ID (http://crispid.gbiomed.kuleuven.be/ (accessed on 20 October 2022) [32]. The parameters for analyzing the chromatogram files were chosen following the instructions of each of the tools.

## 4. Conclusions

Our proposed workflow produces results of the same quality as the old workflow, but in 1 instead of 3 days and with approximately 35 times lower costs. Most of the time is saved by not including overnight steps to grow bacteria, while the bulk of the costs are cut by omitting the expensive step of cloning amplicons into plasmids and sending fewer samples for sequencing. The new workflow also consists of fewer steps, reducing the time spent on genotyping as well as the risk of cross contamination and mistakes. Furthermore, we showed that the automated sequence analysis tools are mostly accurate and can be easily used to detect the CRISR-Cas9-induced mutations. Even though some labs have already adopted or partially adopted our proposed workflow, the advantages detailed here should convince any academic or commercial lab currently using a more time-consuming and expensive method for the very common task of genotyping to switch over immediately.

## Figures and Tables

**Table 1:** Mutation detection accuracy of various online available tools.

No.	Lines	pJet Cloning	DSDecodeme	ICE Software	DECODER v3.0	CRISPR-ID
1	zas-A1_gRNA1	Biallelic mutation (HE)	correct	Single allele correct	correct	correct
2	zas-A3_gRNA1	Homozygote (HO)	correct	correct	correct	correct
3	zas-B1_gRNA1	Biallelic mutation (HE)	correct	correct	correct	not correct
4	zas-B2_gNA1	Biallelic mutation (HE)	correct	correct	correct	not correct
5	zas-B3_gRNA1	Homozygote (HO)	correct	correct	correct	correct
6	zas-B4_gRNA1	Homozygote (HO)	correct	correct	correct	correct
7	zas-D_gRNA1	No mutation	correct	correct	correct	not correct
8	zas-E_gRNA1	Monoalleleci (HE)	correct	correct	correct	not correct
9	zas-F_gRNA1	Monoallelic (HE)	Single allele correct	Single allele correct	Single allele correct	not correct
10	WT_gRNA1	No mutation	correct	correct	correct	correct
11	zas-A1_gRNA2	Biallelic mutation (HE)	correct	correct	correct	correct
12	zas-A3_gRNA2	Homozygote (HO)	correct	correct	correct	correct
13	zas-B1_gRNA2	Biallelic mutation (HE)	correct	correct	correct	correct
14	zas-B2_gNA2	Biallelic mutation (HE)	correct	correct	correct	correct
15	zas-B3_gRNA2	Homozygote (HO)	correct	correct	correct	correct
16	zas-B4_gRNA2	Homozygote (HO)	correct	correct	correct	correct
17	zas-D_gRNA2	No mutation	correct	correct	correct	correct
18	zas-E_gRNA2	Biallelic mutation (HE)	decoding failed	correct	correct	correct
19	zas-F_gRNA2	Homozygote (HO)	correct	correct	correct	correct
20	WT_gRNA2	No mutation	correct	correct	correct	correct

## Data Availability

All data are available in the main text or the Supplementary Materials.

## Supplementary Materials

The following supporting information can be downloaded at: https://www.mdpi.com/article/10.3390/plants12112189/s1, Figure S1: Chromatogram of sequenced direct PCR products. (a) Detailed sequencing results around gRNA1 target site showing the original sequence (WT); homozygous insertion (A3, B3, B4); biallelic insertion (B1, B2, F); heterozygous insertion (E); and a regenerated plant without any mutation (D). (b) Detailed sequencing results around gRNA2 target site showing the original sequence (WT); homozygous insertion (*B4*, *F*); biallelic insertion (*A1*, *B1*, *B2*, *E*); and a regenerated plant without any mutation (D); Table S1: Comparative analysis of different Plant Direct PCR kits in terms of cost, target amplifications and other parameters.
