# Supplementary material for: A Fast and Cost-Effective Genotyping Method for CRISPR-Cas9-Generated Mutant Rice Lines
Source: Plants (Basel). 2023 May 31;12(11):2189. doi: 10.3390/plants12112189 (PMC10255347; doi:10.3390/plants12112189)
Supplement: Supplementary file 1 [file plants-12-02189-s001.zip › plants-2343825- Figure S1.pdf]

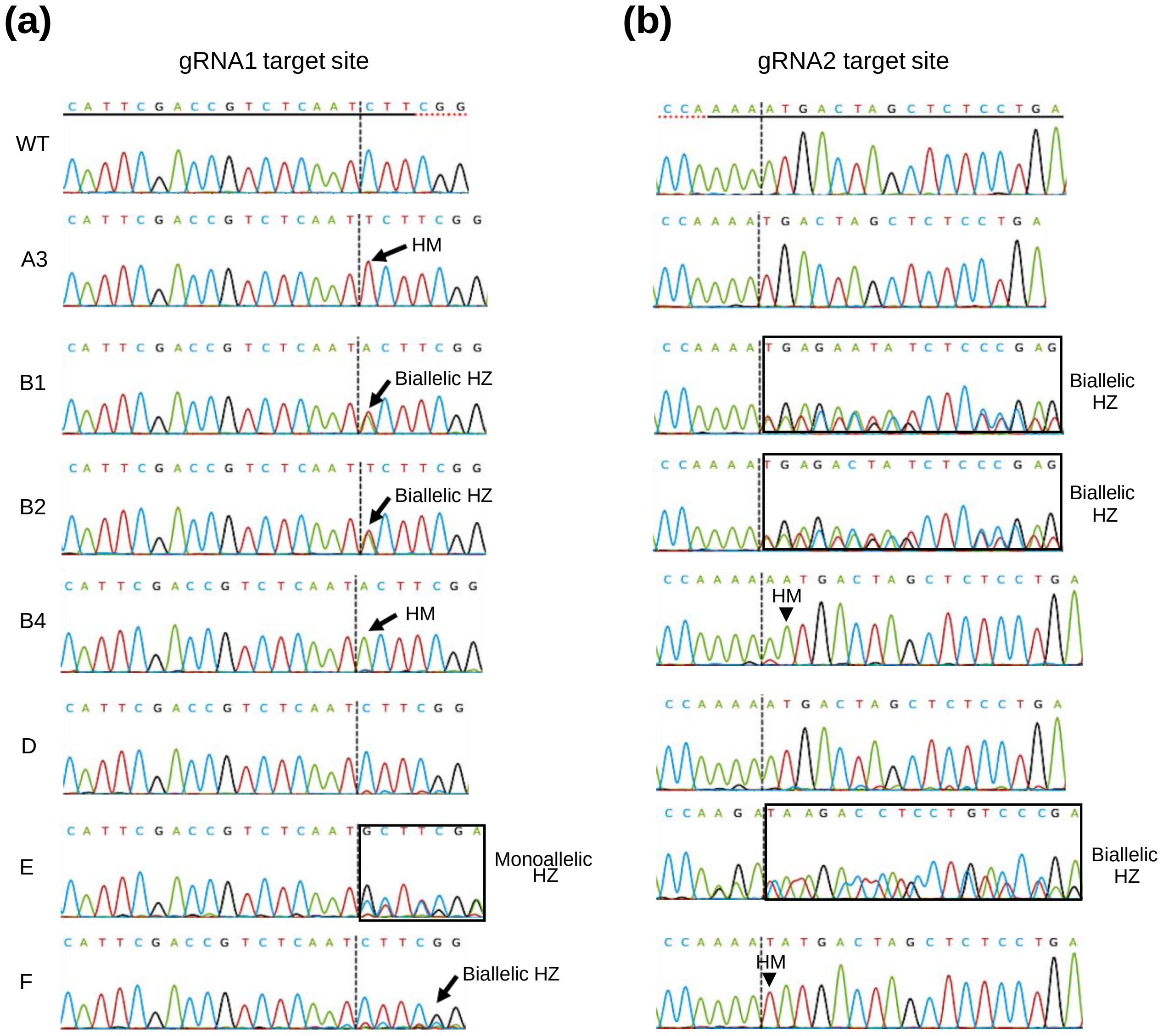

Figure S1. Chromatogram of sequenced direct PCR products. a) Detailed sequencing results around gRNA1 target site showing the original sequence (WT); homozygous insertion (A3, B3, B4); biallelic insertion (B1, B2, F); heterozygous insertion (E); and a regenerated plant without any mutation (D). b) Detailed sequencing results around gRNA2 target site showing the original sequence (WT); homozygous insertion (*B4*, *F*); biallelic insertion (*A1*, *B1*, *B2*, *E*); and a regenerated plant without any mutation (D).
