# Supplementary material for: A Fast and Cost-Effective Genotyping Method for CRISPR-Cas9-Generated Mutant Rice Lines
Source: Plants (Basel). 2023 May 31;12(11):2189. doi: 10.3390/plants12112189 (PMC10255347; doi:10.3390/plants12112189)
Supplement: Supplementary file 1 [file plants-12-02189-s001.zip › plants-2343825- Table S1.pdf]

Table S1. Comparative analysis of different Plant Direct PCR kits in terms of cost, target amplifications and other parameters.

Name of the Kits	Price	Digestion buffer	PCR-ready mixture	Target amplif. size	DNA ladder for Gel run	Control primer
KAPA3G Plant PCR Kit	467.95$ per 250 rxn	No	Yes	up to 5 kb	No	No
Forgene Plant Leaf Direct PCR Plus Kit	N.D yet per 250 rxn	Yes	Yes	up to 1 kb	No	No
Phire Plant Direct PCR Kit	110$ per 250 rxn	Yes	Yes	up to 3.5 kb	Yes	Yes
